# Long‐Term Stability of Spatial Distribution and Peak Dynamics of Subthalamic Beta Power in Parkinson's Disease Patients

**DOI:** 10.1002/mds.30169

**Published:** 2025-03-18

**Authors:** Jennifer K. Behnke, Robert L. Peach, Jeroen G.V. Habets, Johannes L. Busch, Jonathan Kaplan, Jan Roediger, Varvara Mathiopoulou, Lucia K. Feldmann, Moritz Gerster, Juliette Vivien, Gerd‐Helge Schneider, Katharina Faust, Patricia Krause, Andrea A. Kühn

**Affiliations:** ^1^ Movement Disorders and Neuromodulation Unit, Department of Neurology Charité University Medicine Berlin Germany; ^2^ Berlin Institute of Health (BIH) Berlin Germany; ^3^ Department of Brain Sciences Imperial College London London UK; ^4^ UK Dementia Research Institute Imperial College London London UK; ^5^ Department of Neurology University Hospital Würzburg Würzburg Germany; ^6^ Einstein Center for Neurosciences Berlin Berlin Germany; ^7^ NeuroCure Clinical Research Centre Charité University Medicine Berlin Germany; ^8^ Research Group Neural Interactions and Dynamics, Department of Neurology Max Planck Institute for Human Cognitive and Brain Sciences Leipzig Germany; ^9^ Berlin School of Mind and Brain Berlin Germany; ^10^ Department of Neurosurgery Charité University Medicine Berlin Germany; ^11^ German Center for Neurodegenerative Diseases (DZNE) Berlin Germany

**Keywords:** Parkinson's disease, local field potentials, deep brain stimulation, beta band oscillations, subthalamic nucleus

## Abstract

**Background:**

Subthalamic beta oscillations are a biomarker for bradykinesia and rigidity in Parkinson's disease (PD), incorporated as a feedback signal in adaptive deep brain stimulation with potential for guiding electrode contact selection. Understanding their longitudinal stability is essential for successful clinical implementation.

**Objectives:**

We aimed to analyze the long‐term dynamics of beta peak parameters and beta power distribution along electrodes.

**Methods:**

We recorded local field potentials from 12 channels per hemisphere of 33 PD patients at rest, in a therapy‐off state at two to four sessions (0, 3, 12, 18–44 months) post‐surgery. We analyzed bipolar beta power (13–35 Hz) and estimated monopolar beta power in subgroups with consistent recordings.

**Results:**

During the initial 3 months, beta peak power increased (*P* < 0.0001). While detection of high‐beta peaks was more consistent, low‐ and high‐beta peak frequencies shifted substantially in some hemispheres during all periods. Spatial distribution of beta power correlated over time. Maximal beta power across segmented contact levels and directions was significantly stable compared with chance and increased in stability over time. Active contacts for therapeutic stimulation showed consistently higher normalized beta power than inactive contacts (*P* < 0.0001).

**Conclusions:**

Our findings indicate that beta power is a stable chronic biomarker usable for beta‐guided programming. For adaptive stimulation, high‐beta peaks might be more reliable over time. Greater stability of beta power, center frequency, and spatial distribution beyond an initial stabilization period suggests that the microlesional effect significantly impacts neuronal oscillations, which should be considered in routine clinical practice when using beta activity for automated programming algorithms. © 2025 The Author(s). *Movement Disorders* published by Wiley Periodicals LLC on behalf of International Parkinson and Movement Disorder Society.

Oscillatory beta band activity (13–35 Hz) in the subthalamic nucleus (STN) of Parkinson's disease (PD) patients is a well‐characterized biomarker, with its intensity correlating with bradykinetic and rigid symptoms.^1^, ^2^ Both dopaminergic medication and neuromodulation have been observed to suppress beta activity, which aligns with motor improvement.^3^, ^4^, ^5^ Consequently, beta power has become a feedback signal in adaptive deep brain stimulation (aDBS) studies^6^, ^7^ and holds potential for optimizing DBS programming.^8^, ^9^, ^10^, ^11^, ^12^ A recent study in a small PD cohort demonstrated that beta‐guided DBS programming was non‐inferior to clinically guided programming regarding short‐term motor outcomes,^13^ while taking a fraction of time, highlighting the potential of algorithmic beta‐guided optimal contact selection.

A large body of research describes beta power during the acute postoperative period. Recent technological advancements have enabled researchers to derive local field potentials (LFPs) from chronically implanted leads, permitting in‐depth studies of the longitudinal behavior of neural biomarkers.^14^ The long‐term stability of beta power is relevant for both advanced closed‐loop stimulation techniques and beta‐guided DBS programming algorithms. Previous studies have shown that beta activity and Parkinsonian motor symptoms maintained a stable correlation,^15^ while dopaminergic medication and DBS have shown to consistently suppress beta activity over time.^15^, ^16^ More recently, a study reported the reliable detection of peaks in multiple frequency bands across three clinical visits within the first 3 months post‐surgery.^17^ The stability of peak parameters is particularly relevant as current closed‐loop stimulation strategies depend on selecting a frequency range centered on the beta peak for adapting chronic stimulation.^18^ Bipolar LFPs from directional leads capture neural oscillations from multiple distinct recording sites, offering rich spatial insights. The stability of the spatial distribution of beta oscillations along electrode contacts is particularly important for beta‐guided DBS programming.

Our study addresses two primary research questions that are of immediate relevance for clinical application. The first pertains to the longitudinal dynamics of beta peak center frequency (CF) and power to determine whether the peak remains within a frequency range of interest for titrating adaptive stimulation over time. Our second question delves into the stability of the spatial distribution of recorded beta power over time that forms the basis for beta‐guided contact selection in PD patients. We explored these long‐term dynamics of subthalamic beta oscillations in 33 PD patients, spanning up to 44 months post‐implantable pulse generator (IPG) implantation.

## 1 Methods

### 1.1 Participants

This study was approved by the Charité‐Universitätsmedizin Berlin ethics committees (EA2/256/20) and adhered to the Declaration of Helsinki. All participants provided written informed consent prior to participation.

**FIG. 1:**
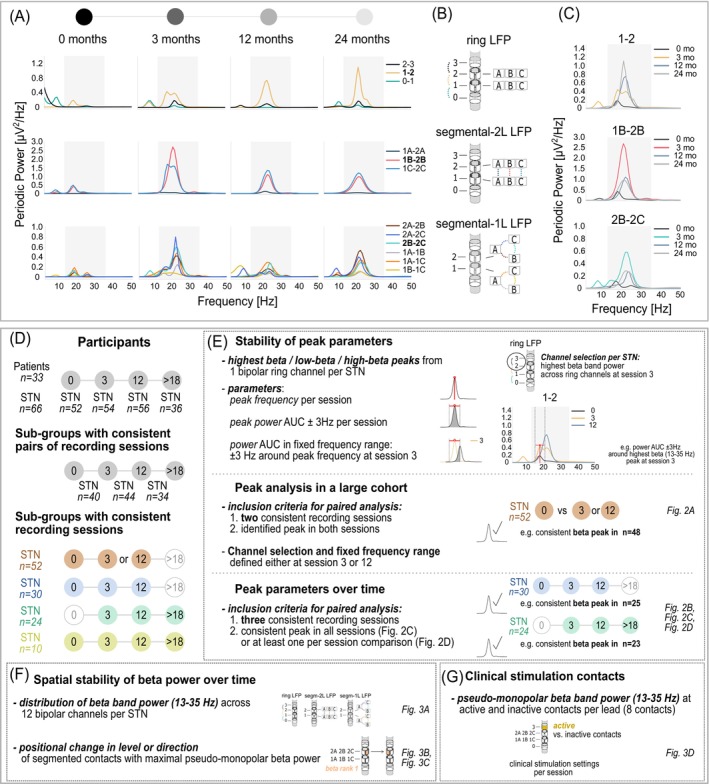
Methods (A) Representative example (patient 8, left hemisphere) of periodic power spectra (beta range in grey) recorded across four post‐surgery sessions. (B) Bipolar local field potentials (LFPs) from the same directional lead were grouped into ring, segmental‐2 L and segmental‐1 L LFP channels. (C) For each group, the channel with maximal beta power at 3‐mo follow‐up (FU) (eg, channel 1–2 in the ring LFP group) was selected for the peak analysis. Power spectra for these channels are shown across sessions (spectra at 3 months in color). (D) A total of 33 patients (n = 66 subthalamic nucleus [STN]) participated, with subgroups defined by consistent pairs or three to four consistent recording sessions (sample sizes illustrated). (E) Long‐term dynamics of peak parameters were analyzed using selected channels and extracted parameters (via *specparam*) in a large cohort (middle panel) with two consistent recording sessions and subgroups with three consistent recording sessions (lower panel). (F) Spatial stability of beta power was analyzed across 12 bipolar channels and positional changes of maximal beta power (eg, beta rank 1 in orange) were investigated using pseudo‐monopolar estimates. (G) Pseudo‐monopolar beta power was analyzed at active and inactive contacts based on clinical stimulation settings per session (eg, contact 3 in yellow). Corresponding figure numbers for each analysis are shown on the right.

Thirty‐three PD patients (11 female) with bilateral subthalamic DBS electrodes (n = 66) were included (clinical details in Table 1; lead localizations in Fig. S4). Participants averaged 62.6 ± 8.3 years (mean ± SD) in age and had a mean disease duration of 10.1 ± 4.5 years. The preoperative International Parkinson and Movement Disorder Society‐Unified Parkinson's Disease Rating Scale‐Part III (Motor Examination) (MDS‐UPDRS‐III) score (medication off) averaged 51.3 ± 16.1. All participants underwent implantation of B33005 “SenSight” directional electrodes (Fig. 1B) and bidirectional Percept™ IPGs (Medtronic, Minneapolis, MN, USA) at the Charité‐Universitätsmedizin Berlin as previously described.^19^

**TABLE 1:** Clinical and demographic details of 33 Parkinson's disease patients

Patient ID	Sex	Age (years)	Disease duration (years)	PD‐phenotype	Symptom dominant side	Medication effect pre‐surgery (MDS‐UPDRS‐III off/on)	Stimulation effect at 12 months (MDS‐UPDRS‐III med‐off, stim‐off/on)	Therapy‐off post‐surgery MDS‐UPDRS‐III per available session	Session (months post‐surgery)
1	f	70	11	M	Right	49/30	44/16	X‐44‐42	3‐12‐44
2	f	43	2	A/R	Left	36/11	19/13	25‐19‐33	3‐12‐18
3	f	71	13	TD	Right	42/7	51/29	41‐51‐36	3‐12‐18
4	m	73	15	A/R	Left	67/43	56/27	61‐36‐56‐59	0‐3‐12‐18
5	m	69	18	A/R	Right	40/25	41/30	37‐42‐41	0‐3‐12
6	m	70	5	M	Right	24/13	31/23	44‐18‐31‐59	0‐3‐12‐36
7	m	58	15	A/R	Left	69/39	38/35	47‐38‐45	0‐12‐24
8	f	65	6	M	Right	68/22	30/20	34‐41‐30‐29	0‐3‐12‐18
9	m	55	4	A/R	Right	69/29	48/33	34‐43‐48‐50	0‐3‐12‐24
10	f	72	12	A/R	Right	43/25	X/X	36‐50	0‐3
11	m	45	7	A/R	Right	76/33	X/X	27‐64	0‐3
12	m	57	6	M	Right	45/13	57/24	45‐57‐63	3‐12‐18
13	f	68	10	A/R	Left	32/12	X/X	38‐38	0‐3
14	m	57	10	A/R	Right	74/42	49/32	56‐43‐56	3‐12‐24
15	m	68	8	A/R	Right	44/16	54/24	42‐54‐64	3‐12‐18
16	f	71	12	M	Right	31/17	31/17	14‐31‐34	0‐12‐18
17	m	57	9	A/R	Left	33/19	35/31	33‐35‐40	0‐12‐18
18	m	66	4	M	Left	58/32	53/31	37‐53	0‐12
19	f	67	7	TD	Left	51/23	40/28	27‐40‐50	3‐12‐18
20	m	53	15	A/R	Right	64/27	52/32	28‐52‐62	0‐12‐18
21	m	61	6	M	Right	33/15	40/27	44‐40‐50	0‐12‐18
22	m	74	20	A/R	Left	52/18	30/23	31‐43‐30‐25	0‐3‐12‐24
23	f	54	8	M	Left	42/27	X/X	49‐51‐63	0‐3‐24
24	m	65	10	A/R	Left	76/21	59/50	35‐58‐59	0‐3‐12
25	m	53	10	A/R	Right	34/13	35/17	15‐59‐36	0‐3‐12
26	f	70	17	TD	Right	52/10	49/32	36‐X‐49	0‐3‐12
27	m	52	14	A/R	Left	74/16	X/X	10–19	0‐3
28	m	61	13	M	Right	65/24	27/12	21‐36‐27	0‐3‐12
29	f	64	4	A/R	Right	31/13	38/30	25‐41‐38	0‐3‐12
30	m	70	6	M	Left	73/36	49/35	44‐47‐49	0‐3‐12
31	m	59	11	A/R	Equal	40/30	36/22	30‐30–36	0‐3‐12
32	m	57	15	A/R	Left	58/39	47/29	34‐60‐47	0‐3‐12
33	m	75	12	M	Right	49/24	31/19	47‐44‐31	0‐3‐12

Each patient participated in at least two of four possible recording sessions: 0‐mo (n = 26) in a range of 0–12 days after deep brain stimulation (DBS) lead implantation, 3‐mo follow‐up (FU) (n = 27), 12‐mo FU (n = 23), and/or >18‐mo FU (n = 18) in a range of 18–44 months post‐surgery.

Abbreviations: PD, Parkinson's disease; MDS‐UPDRS‐III, International Parkinson and Movement Disorder Society‐Unified Parkinson's Disease Rating Scale‐Part III (Motor Examination); m, male; f, female; M, mixed; A/R, akinetic‐rigid; TD, tremor‐dominant; X, not available.

### 1.2 Stimulation Parameters

Stimulation parameters were optimized during scheduled visits at 3‐ and 12‐months with adjustments as needed between and after these visits during outpatient consultations. Final or currently in use DBS settings were documented for each follow‐up (FU) session after the first optimization at 3 months (Table 2).

**TABLE 2:** Clinically optimal stimulation parameters and contacts with maximal pseudo‐monopolar beta power

Patient ID	Sessions (months post‐surgery)	Stimulation parameters LSTN	Stimulation parameters RSTN	Stimulation pulse width, frequency	Contact with maximal pseudo‐monopolar beta power LSTN/RSTN
1	3	1C−2A−, 1.3 mA	1A− 1.2 mA	60 μs, 130 Hz	2A/2A
	12	2−3−, 1.05 mA	2–3−, 1.05 mA	40 μs, 100 Hz	1C/2C
	44	3−, 0.7 mA	3−, 0.7 mA	40 μs, 130 Hz	2A/2C
2	3	1−, 1.3 mA	0−, 1.2 mA	60 μs, 130 Hz	1A/0
	12	1−, 1.5	0−, 1.5 mA	60 μs, 130 Hz	1A/1A
	18	mA 1−2−, 0.7 mA	1−, 1.4 mA	60 μs, 130 Hz	1A/1C
3	3	1−, 2.5 mA	2−, 3.4 mA	60 μs, 130 Hz	1C/1A
	12	0− 1B−, 1.9 mA	0− 1B−, 2.1 mA	60 μs, 130 Hz	1C/1A
	18	0− 1B−, 1.6 mA	0− 1B−, 2.3 mA	60 μs, 125 Hz	1C/1A
4	3	1−, 3.3 mA	1−, 3.8 mA	40 μs, 100 Hz	1A/0
	12	1−, 4.7 mA	1−, 4.3 mA	40 μs, 90 Hz	1A/2A
	18	1−, 4.7 mA	1−, 4.3 mA	40 μs, 90 Hz	1A/2C
5	3	1A− 1B−, 2.5 mA	1−, 2.2 mA	60 μs, 130 Hz	1A/1A
	12	2−, 2.5 mA	2−, 2.3 mA	60 μs, 125 Hz	1A/2A
6	3	1A− 1B−, 3 mA	2A− 2B−, 2.9 mA	60 μs, 125 Hz	1A/1A
	12	1A− 1B−, 5.1 mA	2A− 2B−, 4.5 mA	60 μs, 125 Hz	1A/1A
	36	1−, 2.8 mA	1−, 2.7 mA	40 μs, 110 Hz	1A/1A
7	12	1C− 2C−, 2.5 mA	1−, 2.6 mA	60 μs, 125 Hz	1A/2A
	24	1C− 2C−, 2.5 mA	1−, 2.6 mA	60 μs, 125 Hz	1A/2A
8	3	IL 1–2+/3−, 0.9 mA	IL 2A− 2B−/1A− 2−, 1.4 mA	40 μs, 90 Hz	1A/2A
	12	2C−, 0.9 mA	1C− 2C−, 1.4 mA	60 μs, 125 Hz	1A/1A
	18	2C−, 2 mA	1A− 2A−, 1.9 mA	60 μs, 125 Hz	1C/2A
9	3	2C−, 1.9 mA	2C−, 1.9 mA	60 μs, 130 Hz	2B/2B
	12	2−, 2.8 mA	2−, 2.8 mA	60 μs, 130 Hz	1C/2B
	24	2B− 2C−, 4 mA	2A− 2B−, 4.2 mA	60 μs, 130 Hz	2B/3
10	3	1−, 1.5 mA	1−, 1.4 mA	60 μs, 125 Hz	2B/1C
11	3	2B− 2C−, 1.2 mA	2−, 1.1 mA	60 μs, 130 Hz	2C/1B
12	3	1A− (40%) 1B− (100%) 2A− (60%) 2B− (100%), 2.3 mA	2A− (120%) 2B− (80%), 1.9 mA	60 μs, 125 Hz	2A/1B
	12	2A− 2B− 3−, 2.8 mA	2A− 2B− 3−, 3.1 mA	60 μs, 125 Hz	2A/2B
	18	2A− 2B− 3−, 2.8 mA	2A− 2B− 3−, 3.1 mA	60 μs, 125 Hz	2A/2B
13	3	2−, 2.7 mA	2−, 1.6 mA	60 μs, 125 Hz	1B/2A
14	3	2−, 1.7 mA	2−, 2.2 mA	60 μs, 130 Hz	2C/1B
	12	2−, 3,4 mA	2−, 2.7 mA	60 μs, 130 Hz	2B/1B
	24	2−, 3.4 mA	2−, 2.7 mA	60 μs, 130 Hz	2B/2A
15	3	2−, 1.3 mA	1−, 1.3 mA	60 μs, 130 Hz	2C/2B
	12	2−, 2 mA	1−, 2 mA	60 μs, 110 Hz	2C/2B
	18	2−, 2.4 mA	1−, 2.5 mA	60 μs, 80 Hz	2A/2B
16	12	1–2−, 1.5 mA	1–2−, 1.2 mA	60 μs, 90 Hz	2B/2B
	18	1–2−, 1.75 mA	1–2−, 1.5 mA	60 μs, 125 Hz	2B/2C
17	12	1−, 2.8 mA	0−, 2.8 mA	40 μs, 130 Hz	1B/1C
	18	1–, 2.8 mA	0−, 2–8 mA	40 μs, 130 Hz	1B/1C
18	12	1−, 1 mA	1−, 3 mA	60 μs, 130 Hz	2A/1A
19	3	2−, 1.7 mA	2−, 1.7 mA	60 μs, 130 Hz	1A/1A
	12	2B− 2C− 3−, 2.3 mA	2B− 2C− 3−, 2.6 mA	60 μs, 110 Hz	1A/1B
	18	2−, 1.5 mA	2−, 1.7 mA	60 μs, 180 Hz	1A/1A
20	12	1+ 2–3+, 2.5 mA	1B− 2B−, 5.7 mA	60 μs, 130 Hz	2C/2B
	18	1+ 2– 3+, 3 mA	1B− 2B−, 5.7 mA	60 μs, 130 Hz	2C/1A
21	12	1−, 2.5 mA	1−, 2.5 mA	60 μs, 100 Hz	2C/1C
	18	1−, 2 mA	1−, 2 mA	60 μs, 130 Hz	2C/1C
22	3	1−, 1.8 mA	2−, 1.6 mA	60 μs, 125 Hz	1A/1B
	12	1−, 2.2 mA	2−, 2.6 mA	60 μs, 125 Hz	1A/1B
	24	1−, 2.6 mA	2−, 2.8 mA	60 μs, 125 Hz	1A/2B
23	3	2−, 1.8 mA	2−, 2.1 mA	60 μs, 90 Hz	1B/2A
	24	IL 2−, 1.2/0.9 mA	2−, 1.3 mA	50 μs, 125 Hz; LSTN IL 125/95 Hz	1A/1A
24	3	1−, 3.5 mA	2−, 3.2 mA	60 μs, 125 Hz	2A/2A
	12	2−, 2.7 mA	2−, 2.7 mA	60 μs, 90 Hz	
25	3	1−, 2.4 mA	1−, 2.4 mA	60 μs, 130 Hz	2C/2B
	12	1−, 2.4 mA	1−, 2.3 mA	60 μs, 85 Hz	
26	3	2−, 2.3 mA	2−, 2.6 mA	60 μs, 90 Hz	2A/2A
	12	2–3−, 1.9 mA	2–3−, 1.7 mA	60 μs, 100 Hz	
27	3	1−, 2.5 mA	2−, 2.5 mA	60 μs, 125 Hz	1C/1C
28	3	1−, 1.5 mA	1−, 1.9 mA	60 μs, 125 Hz	2C/2A
	12	1−, 1.7 mA	1−, 2.0 mA	60 μs, 125 Hz	1A/1B
29	3	3−, 0.5 mA	3–1+, 1.1 mA	L60/R40 μs	2C/2B
	12	3−, 1.4 mA	2A− 2B−, 0.9 mA	60 μs, 130 Hz	1A/2C
30	3	1−, 2.9 mA	1−, 2.4 mA	60 μs, 180 Hz	2C/2C
	12	2−, 2.8 mA	2−, 2.6 mA	60 μs, 180 Hz	2C/2A
31	3	1−, 2.0 mA	1−, 1.7 mA	60 μs, 125 Hz	2A/1C
	12	1−, 1.8 mA	1−, 1.8 mA	60 μs, 130 Hz	2A/1C
32	3	2−, 1.5 mA	2−, 1.5 mA	60 μs, 130 Hz	2C/1C
	12	2−, 1.5 mA	2−, 1.5 mA	60 μs, 130 Hz	2C/2B
33	3	1−, 2.4 mA	2−, 2.2 mA	60 μs, 130 Hz	1A/2A
	12	1−, 2.6 mA	2−, 2.6 mA	60 μs, 130 Hz	1C/2C

This table shows the clinically optimal stimulation parameters for each patient at each participated session as well as the segmented contacts aligning with maximal pseudo‐monopolar beta power per deep brain stimulation (DBS) lead. Since stimulation parameters were optimized at 3 months post‐surgery, stimulation parameters at session 0‐months are not presented. Patient 8 (bilaterally, session 3) and 23 (LSTN, session 24) used an interleaving stimulation program (IL) and patient 20 (LSTN, session 12 and 18) and 29 (RSTN, session 3) used a bipolar stimulation program.

Abbreviations: LSTN, subthalamic nucleus of the left hemisphere; RSTN, subthalamic nucleus of the right hemisphere.

### 1.3 Recording Procedure

Neurophysiological recordings were conducted while participants were seated at rest in a medication‐off (≥12 h) and stimulation‐off state (≥30 min).^20^ Each patient attended at least two of four possible LFP sessions at 0‐ (0–12 days after lead implantation), 3‐, 12‐, and/or >18‐months post‐surgery (session details in Table 1). Due to limited numbers, recordings from 18 to 44 months were grouped (>18‐mo FU). Since session participation varied between patients, analyses of subgroups with consistent session participation were performed (see Table S3 and Fig. 1D for details). A subgroup with four consistent recordings (n = 10 STN) was reported but not further analyzed due to the small size.

### 1.4 Data Acquisition

We recorded neural data from 12 bipolar channels per lead using the IPG's “BrainSense Survey” (BSSU) mode with 250 Hz sampling rate, 1 Hz high‐pass filter and ~ 20 s duration. Each lead has eight contacts across four levels (Fig. 1B): two ring contacts (levels 0 and 3) and six segmented contacts in the middle levels (A, B, C). LFPs were recorded in three bipolar groups: adjacent contact levels (ring: 0–1, 1–2, 2–3), inter‐level segments (segmental‐2 L: 1A‐2A, 1B‐2B, 1C‐2C), and segments of the same level (segmental‐1 L: 1A‐1B, 1A‐1C, 1B‐1C, 2A‐2B, 2A‐2C, 2B‐2C). Recordings were exported to the JSON format for offline analysis.

### 1.5 LFP Processing

**FIG. 2:**
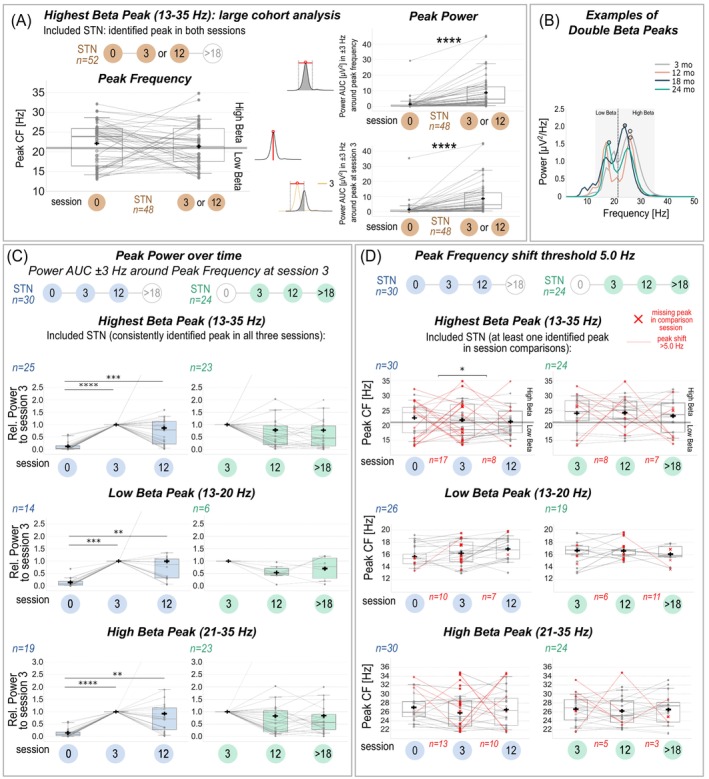
Beta peak parameters over time. (A) Peak parameters of the highest beta peak (13–35 Hz) identified in both sessions 0‐ and 3‐/12‐mo follow‐up (FU) (n = 48). Left: Peak frequencies often shift between sessions (50% >5 Hz), but without a consistent directional trend. The grey horizontal line at 21 Hz marks the boundary between low‐ and high‐beta ranges. Right: Peak power significantly increases (*P* < 0.0001) for both power area under the curve (AUC) ± 3 Hz around the peak center frequency (CF) (top) and AUC in a fixed frequency range ± 3 Hz around the peak CF at 3‐/12‐mo FU (bottom). (B) Examples of double peaks in the beta range (shaded grey) in patient 12 (right hemisphere, channel 2–3) showing a switch of the highest peak from the high‐ to the low‐beta range at 24 months (green). The dashed line marks the boundary between low‐ and high‐beta bands. (C, D) Subgroup analyses of one selected channel per subthalamic nucleus (STN) with consistent recording sessions: blue (0‐3‐12), green (3‐12‐>18). Channels with consistently identified peaks in all sessions (C) or at least one identified peak per session comparison (D) were included. Peak power (AUC in a fixed frequency range defined at and normalized to 3 months) (C) and peak frequency (D) are illustrated for each session in the full beta (top), low‐beta (middle) and high‐beta band (bottom). Lines connect peaks from the same hemisphere. Only in D, missing peaks are marked with a red cross at the compared peak CF from the other session. Red lines highlight CF shifts >5 Hz. The number of channels with significant CF shifts (>5 Hz) or peak disappearance is shown below in red (eg, of 30 hemispheres, the largest beta peak shifted >5 Hz between sessions 0‐ and 3‐mo FU in n = 17 hemispheres). The grey line at 21 Hz in the top panel marks the border between the low‐ and high‐beta boundary. Black crosses in boxplots indicate the mean.

Raw LFPs were visually screened for movement or electrocardiogram (ECG) artifacts, which were removed using Independent Component Analysis (ICA) (Fig. S1). Spectrograms were computed with short‐time Fourier transforms using 1 s Hanning windows and 50% overlap. Power spectral density was averaged over the full recording duration. The spectral parameterization software (*specparam*, formerly *FOOOF*: parameter details in Fig. S2)^21^ was used to isolate periodic components^22^, ^23^ and identify peak CF and power. Analyzed features included periodic beta band power (13–35 Hz), peak CF, peak power (area under the curve [AUC] ± 3 Hz of the peak CF), and power in a fixed frequency range (AUC ± 3 Hz of the 3‐ or 12‐mo FU peak CF) for beta (13–35 Hz), low‐beta (13–20 Hz), and high‐beta ranges (21–35 Hz). For the peak parameter analysis (Fig. 1E), the ring LFP channel with highest beta band power at 3‐mo FU (or 12‐mo FU in Fig. 2A) was selected, assuming greater stability after the early postoperative period. Beta power was detected in at least one channel per hemisphere. Pseudo‐monopolar beta power (13–35 Hz) aligning to individual contacts was estimated by our newly developed method using Euclidean distance weighting (see details in Fig. S3, Tables S1 and S2) to assess the positional change of maximal beta power and the clinical relevance in contact selection (Fig. 1F,G).

### 1.6 Statistics

Detailed inclusion criteria for each analysis are provided in the Statistics section of the Supplementary Material.

#### 1.6.1 Peak Parameter Analysis

Paired Wilcoxon signed‐rank tests were used for subgroups with two consistent sessions (Fig. 2A). For subgroups with three consistent sessions, either Friedman (with post‐hoc Wilcoxon signed‐rank) or repeated‐measures ANOVA tests (r‐m ANOVA with post‐hoc paired *t*‐tests) were applied based on data normality. Holm correction adjusted for multiple comparisons (Fig. 2C, Fig. S6B). Peak frequency shifts (>2.5 or >5 Hz) or missing peaks were classified as substantial shifts and analyzed across periods using Wilcoxon tests for binomial classifications (shift vs. no shift) (Fig. 2D, Fig. S6A). These thresholds were chosen due to their potential impact on aDBS performance. Shifts >2.5 Hz could partially, and >5.0 Hz could entirely, displace peaks from a fixed frequency range (±2.5 Hz). Additional analyses are detailed in the Supplementary Material (Fig. S5).

#### 1.6.2 Spatial Distribution Analysis

**FIG. 3:**
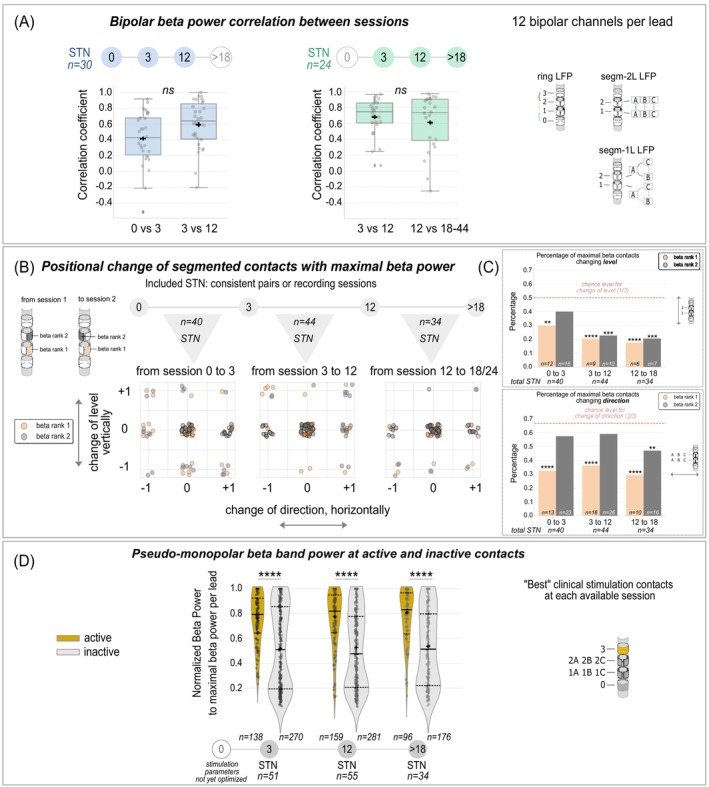
Spatial distribution of beta oscillations over time. (A) Mean Spearman's correlation between ranked beta power from 12 bipolar channels (ring and segmental) at consistent sessions across hemispheres from the “early” subgroup (blue, n = 30 subthalamic nucleus [STN]) and the “late” subgroup (green, n = 24 STN) show lowest correlation between 0‐ and 3‐mo follow‐up (FU) but no significant differences across session comparisons. (B) Positional change in level (y) and direction (x) of segmented contacts with maximal beta values (beta rank 1: orange, 2: grey) between sessions. An exemplary lead is shown on the left. The positional changes across hemispheres are shown in a simplified coordinate system (y: change in level 1 and 2; x: change in segments A–C). N = number of STN with consistent pairs of recording sessions. 0 = no change, +1 and −1 = change in direction/level. (C) The percentage of maximal beta contacts changing level (top) or direction (bottom) are illustrated for each pair of recording sessions. Beta rank 1 (orange) changes were significantly lower than chance level (red dashed line) for both levels and directions across all periods, while beta rank 2 contacts (grey) fluctuated more in the earlier periods. (D) Pseudo‐monopolar beta power from eight contacts per hemisphere, normalized to maximal beta power within a deep brain stimulation (DBS) lead was higher in active (gold) than inactive (grey) contacts for chronic stimulation after each follow‐up visit at 3‐, 12‐, and >18‐mo FU (session 0‐mo is not shown because stimulation parameters were not yet optimized). Horizontal solid black lines indicate the median. Horizontal dashed lines at the bottom show the first quartile and at the top show the third quartile. Black crosses indicate the mean.

Spearman's rank correlation evaluated the longitudinal stability of ranked bipolar beta power across 12 channels (ring and segmental) per lead. Fisher‐transformed coefficients were compared using paired *t*‐tests (Fig. 3A). Binomial tests evaluated vertical (z: levels 1 and 2) and horizontal (xy: directions A, B, C) shifts of segmented contacts with maximal pseudo‐monopolar beta power (beta rank 1 and 2) relative to chance for each period (Fig. 3B,C). Mann–Whitney U‐tests compared normalized pseudo‐monopolar beta power (relative to maximum per lead) at clinically active versus inactive contacts (including all eight contacts, Fig. 3D).

Data normality was assessed using Shapiro–Wilk tests. Statistical significance: ns *P* ≥ 0.05, * *P* < 0.05, **P < 0.01, ***P < 0.001, ****P < 0.0001. Results were reported as mean ± SD.

### 1.7 Software

Software details are provided in the Supplementary Material.

## 2 Results

### 2.1 Consistency of Peak Identification over Time

The consistency of peak identification over time (via *specparam*; see Supplementary Material) varied across beta ranges (Fig. S7). In the “early” subgroup (n = 30 hemispheres, 0‐, 3‐, and 12‐mo FU), consistent peaks (identified in all three sessions) appeared in 83% of hemispheres in the full beta range, but only 47% in low‐beta and 63% in high‐beta ranges. In the “late” subgroup (n = 24 hemispheres, 3‐, 12‐, and >18‐mo FU), consistent peaks were identified in 96% for full beta and high‐beta ranges but only 25% in low‐beta.

### 2.2 Modulation of Beta Peak Power over Time

Among 52 hemispheres with consistent recordings at 0‐mo FU and either 3‐mo (n = 40) or 12‐mo FU (n = 12), 48 displayed a beta peak (13–35 Hz) in both sessions. Beta peak power significantly increased between 0‐ and 3‐/12‐mo FU for both power metrics: (1) power AUC ± 3 Hz around peak CF (mean difference ± SD: −7.39 ± 8.79, *P* < 0.0001) and (2) AUC in a fixed frequency range ± 3 Hz around the 3‐/12‐mo FU peak CF (−7.29 ± 8.67, *P* < 0.0001; Fig. 2A).

In subgroups with three consistent sessions and consistently identified peaks, both power metrics showed similar results. Hence, only results from power AUC ± 3 Hz of the 3‐mo FU peak CF are reported. Normalized power (relative to 3‐mo FU) significantly increased from 0‐ to 3‐mo FU and stabilized thereafter (Friedman, Fig. 2C).

In the “early” subgroup, relative power at 0‐mo FU (eg, full‐beta: 0.13 ± 0.18) was significantly lower than 3‐mo FU (1.00 ± 0.00 *P* < 0.0001) and 12‐mo FU (0.86 ± 0.96, n = 25, *P* = 0.0002 *Holm‐corrected*). Similar trends occurred in low‐beta (n = 14) and high‐beta ranges (n = 19), without significant differences between 3‐ and 12‐mo FU (*P* ≥ 0.05).

In the “late” subgroup, relative power showed a decreasing trend from 3‐mo FU (1.00 ± 0.00) to later sessions but did not reach significance (eg, 12‐mo FU full‐beta: 0.79 ± 1.03, n = 23, *Holm‐corrected P* = 0.064; low‐beta: 0.54 ± 0.31, n = 6, *Friedman P* = 0.069; high‐beta: 0.83 ± 1.03, n = 23, *Holm‐corrected P* = 0.106). No difference occurred between 12‐ and >18‐mo FU (*P* ≥ 0.05).

### 2.3 Change in Peak Frequency over Time

In 52 hemispheres (n = 48 with consistent beta peaks in two sessions), the highest beta peaks showed no significant directional shifts (0.66 ± 6.96, *P* = 0.461), though 50% exceeded 5 Hz shifts and ranged between 0.05 and 15.67 Hz (Fig. 2A).

In the “early” subgroup with three consistent sessions (Fig. 2D), 57% of the largest beta peaks shifted >5 Hz or disappeared between 0‐ and 3‐mo FU, significantly more than between 3‐ and 12‐mo FU (27%, *P* = 0.020, n = 30). In the “late” subgroup, the highest beta peak shifts occurred in 33% (3‐ and 12‐mo FU) and 29% (12‐ and >18‐mo FU) without significant differences (*P* ≥ 0.05). Shifts were particularly prominent when the highest beta peaks alternated between low‐ and high‐beta ranges in double peaks (see Fig. 2B). Additional analysis comparing absolute peak frequency shifts confirmed these findings (Fig. S5, Table S4).

For low‐beta and high‐beta peaks, shifts >5 Hz or disappearance occurred in all periods without significant differences over time (*P* ≥ 0.05, Fig. 2D). In the “early” subgroup, substantial shifts appeared in 38% of low‐beta (from n = 26) and 43% of high‐beta peaks (from n = 30) between 0‐ and 3‐mo FU, while slightly less between 3‐ and 12‐mo FU (27% and 33%, respectively). In the “late” subgroup, substantial shifts occurred in 32% of low‐beta (from n = 19) and 21% of high‐beta peaks (from n = 24) between 3‐ and 12‐mo FU, while between 12‐ and >18‐mo FU in 58% and 12%, respectively. Shifts >2.5 Hz were more common but followed similar trends (Fig. S6A).

Directional shifts were significant only for consistently identified low‐beta peaks in the “early” subgroup, increasing from 15.65 ± 1.70 (0‐mo FU) to 16.93 ± 1.94 (12‐mo FU, *Friedmann*, *Holm‐corrected P* = 0.040, n = 14; Fig. S6B). No directional shifts occurred in the “late” subgroup (n = 6, *P* = 0.806 *r‐m ANOVA*) or in other beta ranges (*P* ≥ 0.05 *Friedmann*).

### 2.4 Spatial Distribution of Beta Power is Stable and Correlates Over Time

The ranked order of bipolar beta power across 12 channels (ring and segmental LFP) showed positive correlations over time. The lowest correlations were observed in the “early” subgroup (n = 30) between 0‐ and 3‐mo FU (0.41 ± 0.32), though not significantly different from 3‐ and 12‐mo FU correlations (0.59 ± 0.30, *P* = 0.096). The “late” subgroup (n = 24), maintained high correlations across sessions (3–12‐mo FU: 0.68 ± 0.23; 12‐ >18‐mo FU: 0.61 ± 0.34, *P* = 0.69; Fig. 3A).

Beta rank 1 contacts with maximal pseudo‐monopolar beta power (Fig. 3B, orange) were increasingly stable over time on the vertical axis (levels 1 and 2) with significantly fewer positional shifts in their level than expected by chance (50%) across all periods (0–3‐mo FU: 30% from n = 40, *P* = 0.008; 3–12‐mo FU: 20% from n = 44, *P* < 0.0001; and 12‐ to >18‐mo FU: 18% from n = 34, *P* < 0.0001; Fig. 3C, top). Also, on the horizontal plane (directions A, B, C) shifts were significantly lower than chance (67%) across all periods (0–3‐mo FU: 32%, *P* < 0.0001, 3–12‐mo FU: 36%, *P* < 0.0001, 12‐ >18‐mo FU: 29%, *P* < 0.0001; Fig. 3C, bottom). Beta rank 2 contacts were less stable, fluctuating vertically in the earliest period (0–3‐mo FU: 40%, *P* = 0.134), but stabilizing later (3–12‐mo FU: 23%, *P* = 0.0002; 12‐ >18‐mo FU: 21%, *P* = 0.0004). Horizontally, beta rank 2 contacts fluctuated up to 12‐mo (0–3‐mo FU: 57%, *P* = 0.146; 3–12‐mo FU: 59%, *P* = 0.184), and stabilized later (12‐ >18‐mo FU: 47%, *P* = 0.015).

### 2.5 Stimulation Settings Reflect Differences in the Beta Distribution Across Contacts

After excluding hemispheres with bipolar or interleaving stimulation programs (n = 6), remaining hemispheres (3‐mo FU: n = 51, 12‐mo FU: n = 55, >18‐mo FU: n = 34) showed the following distribution of active versus inactive contacts used for chronic stimulation: 3‐mo FU: respectively, n = 138 vs. n = 270, 12‐mo FU: n = 159 vs. n = 281 and >18‐mo FU: n = 96 vs. n = 176 (Fig. 3D). Normalized pseudo‐monopolar beta power (to the maximum within a lead) was significantly higher at active (3‐mo FU: 0.76 ± 0.20, 12‐mo FU: 0.76 ± 0.23, >18‐mo FU: 0.78 ± 0.21) compared with inactive contacts across all sessions (3‐mo FU: 0.53 ± 0.33; 12‐mo FU: 0.51 ± 0.31; >18‐mo FU: 0.53 ± 0.30, *P* < 0.0001). Contacts with maximal beta power were active in 45% of hemispheres (23/51) at 3‐mo FU, 53% (29/55) at 12‐mo FU and 50% (17/34) at >18‐mo FU. When considering at least one of the two contacts with maximal beta power, these percentages increased to 58%, 69% and 68% of hemispheres, respectively.

## 3 Discussion

This study examined the stability of the spatial distribution and peak parameters of subthalamic beta oscillations in 33 PD patients over a maximum of up to 44 months post‐surgery. Our findings indicate significant fluctuations primarily during the early postoperative period with increasing stability observed in the long term.

### 3.1 Stability of Beta Peak Parameters After Surgery and its Relevance for aDBS

In line with the microlesional/stun effect,^24^, ^25^, ^26^ substantial fluctuations in beta peak parameters occurred mainly in the early postoperative period. Beta power significantly increased between 0‐ and 3‐mo FU and stabilized thereafter (Fig. 2A,C). Similarly, beta peak CF fluctuated more in early compared with later periods, particularly for the largest peak in the full beta range (13–35 Hz; Fig. 2D). These fluctuations, including alternations between low‐beta and high‐beta peaks in cases of double beta peaks (Fig. 2A,B,D), emphasize the importance of distinguishing these sub‐beta bands for precise aDBS settings. These early fluctuations are likely related to an acute tissue response (microlesional/stun effect) after lead placement, temporarily improving Parkinsonian symptoms even without stimulation in some patients.^24^, ^25^, ^26^ An impedance mismatch, occurring between the tissue and recording electrode, can impact the amplitude and frequency characteristics of LFP signals, especially at higher frequencies.^27^ Given our results, this effect influences the spectral power in the first session. However, in line with clinical observations and previous findings^24^ our results corroborate the idea that the microlesional effect seems to no longer affect LFPs after an initial period of maximally 3 months post‐implant.

Consistent with previous studies reporting no progression of beta oscillations for up to 3 years,^28^, ^29^, ^30^ we observed no substantial beta power increases beyond stabilization over 44 months. A slight non‐significant decrease in power was observed when defining a fixed frequency range based on the 3‐mo FU recording (Fig. 2C). This effect may be attributed to minor fluctuations in the peak frequency, which led to decreased power AUC within the specified frequency range. Peak power AUC ± 3 Hz around its own peak CF showed a similar but less strong decreasing trend. In line with previous findings,^15^, ^31^ only mild absolute CF shifts were observed on average for both low‐beta (<2 Hz) and high‐beta peaks (2–3 Hz) over time, regardless of peak disappearance (Fig. S5, Table S4).

Together, these findings suggest setting power thresholds for aDBS after a stabilization period of up to 3 months. However, the exact timing of stabilization after surgery remains unclear, as intermediate measurements were not assessed. Long‐term adjustments to the power thresholds, peak frequency, and contact selection may be required to optimize aDBS outcomes. Here, it is interesting to note that in our study high‐beta peaks were more consistently detected (96%) than low‐beta peaks (25%) during long‐term follow‐up (Fig. S7), suggesting that high‐beta peaks may be more reliable for defining optimal aDBS frequency ranges. High‐beta peaks showed no significant directional CF shifts over time, while low‐beta peaks demonstrated a significant CF increase from session 0‐ to 12‐mo FU in a subgroup of patients (n = 14). However, low‐beta peak analysis in late sessions was limited by low numbers (n = 6; Fig. S6B). A key consideration for aDBS is the potential for peaks shifting out of the selected frequency range (± 2.5 Hz). Notably, shifts >5.0 Hz or peak disappearance occurred in 58% of low‐beta peaks and only 12% of high‐beta peaks between sessions 12‐ and >18‐mo FU (Fig. 2D), highlighting the greater stability of high‐beta peaks. However, it remains unclear whether the higher variability or disappearance of low‐beta peaks might be related to fluctuations in patients' medication (eg, residual effects of dopamine agonists with longer half‐lives after 12 h of withdrawal) or to long‐term DBS effects (eg, neuroplastic changes). Further research in larger patient cohorts and more experience with adaptive stimulation will be essential to better understand these factors and develop more precise and informed recommendations for effective therapy.

### 3.2 Maximal Beta Power is a Stable Chronic Biomarker Usable for Beta‐Guided Contact Selection

For beta‐guided DBS programming the spatial stability of maximal beta power is crucial, particularly along the z‐axis for ring stimulation. Our findings show a positive correlation of the ranked order of beta power across all bipolar channels over time (Fig. 3A). Here, the lowest correlation was observed in the early postoperative period between sessions 0‐ and 3‐mo FU, pointing to a higher variability in the early postoperative phase, which is in line with observations in non‐human primates that showed fluctuations in the first 2 weeks post‐implant and subsequent stabilization.^24^

We further observed that segmented contacts with maximal pseudo‐monopolar beta power showed increasing stability along the z‐axis over 44 months (up to 82% of contacts remaining in their level, Fig. 3B,C). This finding extends recent studies, which described constant bipolar contact pairs with LFP peaks along the z‐axis (ring LFP) at multiple visits during the first 3 months^17^ and within the same session.^32^ Our result supports the long‐lasting stability of beta‐guided contact selection for ring stimulation, especially over the long term.

For directional stimulation, the stability of maximal beta power along the horizontal plane is relevant. We found that the segmented contact with highest beta power remains stable in its directionality in most hemispheres (64–71% remaining in the same direction, Fig. 3B,C). However, contacts with second highest beta power showed more fluctuation in their directional position over time. These findings suggest that selecting directional contacts (especially more than one segmented contact) based on beta power may be less reliable and may require longer or repeated LFP recordings. The implications of these horizontal long‐term fluctuations on the therapeutic efficacy of segmented contacts remain unclear and require further investigation.

### 3.3 Potential Use of Beta Power for Active Contact Selection

We have shown that active contacts selected for therapeutic stimulation correspond to higher normalized beta power than inactive contacts at every follow‐up session (Fig. 3D). Inactive contacts, not used for chronic stimulation, showed mostly low, but also high beta values. These contacts might have not consistently been tested for clinical use or the decision not to use certain contacts may have been driven by other factors that are not reflected in beta power, for example, tremor or stimulation‐induced side effects. Between 45% and 69% of contacts with maximal beta power were chosen for therapeutic use. Our findings support the utility of beta power as a relevant but not exclusive tool for DBS programming. This highlights one of the many complexities in introducing a fully automatized DBS programming algorithm, a goal that has become increasingly important as stimulation parameters grow more complex. An example of a data‐driven algorithm is “StimFit”, providing personalized stimulation parameters based on the electrode‐location and neuroimaging‐derived metrics, while considering motor outcome and stimulation‐induced side effects.^33^, ^34^ Neuroimaging‐guided techniques form a good basis for initial DBS programming. However, chronically derived LFPs could complement these automated techniques well by adjusting for long‐term fluctuations. As shown in our study, fluctuations of the spatial distribution of beta oscillations might be especially relevant for directional stimulation. Despite potential benefits of directional stimulation such as higher side effect thresholds and larger therapeutic windows,^35^ our patient cohort demonstrates its underuse with only 21% of hemispheres using directional stimulation at the 12‐mo FU. This underuse is likely due to the time‐consuming programming process. Multimodal DBS programming combining the use of biomarkers both from LFPs as well as imaging and their proportion of valuable information might be an efficient approach in the future.^36^, ^37^

### 3.4 Limitations

Several limitations of this study should be considered. Five patients participated in only two sessions, though most patients (n = 28) participated in at least three sessions and in most main analyses participants with consistent recording sessions were used. Recordings in the BSSU mode are limited to 20 s approximately, which captures only a short window of beta oscillations. Individual short‐term fluctuations of beta activity during the day and from day to day must be considered. Nevertheless, we found a significant stability of beta band parameters over time that was most likely more compromised by the stun effect than short recording durations. Long‐term recordings might also be influenced by impedance changes^24^, ^38^, ^39^ as higher electrode impedances might contribute to noise in the recordings.^40^ However, it has been repeatedly reported that fluctuations of DBS lead impedances did not correlate with long‐term beta activity dynamics, which suggests other chronic processes being involved in longitudinal beta fluctuations.^24^, ^41^

Furthermore, IPG devices used in this study only allowed for bipolar LFPs. Hence, estimations of monopolar beta power in our analyses were based on weighting methods (Fig. S3, Tables S1 and S2) with limitations especially for the ring contacts 0 and 3, for example, due to different impedances compared with segmented contacts. Therefore, in some analyses if reasonable, we used pseudo‐monopolar beta power only for directional contacts and excluded the ring contacts 0 and 3 (Fig. 3C,D). Future technological advancement may overcome this limitation by enabling monopolar power derivation.

## 4 Conclusions

This study is the first to demonstrate in a large PD patient cohort the long‐term stability of beta activity parameters using a chronic sensing IPG available in routine clinical care. During the initial postoperative period up to 3 months, beta power increased, and the spatial distribution showed variability most likely due to the stun effect after electrode implantation. Beyond the early postoperative period, the spatial distribution and peak parameters of subthalamic beta band oscillations showed higher stability, supporting their usability for biomarker‐guided programming and adaptive DBS. Programming of aDBS parameters would thus be recommended only after the stun effect resolves and beta power parameters stabilize as needed for aDBS threshold settings. While peak frequency fluctuations can occur during all periods, high‐beta peaks demonstrate greater consistency and stability than low‐beta peaks, making them a potentially more reliable target for defining aDBS frequency ranges. In the long term, beta power was higher at clinically active contacts as compared with inactive contacts, which further supports the potential for beta‐guided DBS contact selection. Taken together, our findings are relevant information for both future beta‐guided contact selection algorithms and aDBS approaches.

## Author Roles

(1) Research Project: A. Conceptualization, B. Methodology, C. Software, D. Investigation, E. Visualization, F. Data Curation; (2) Statistical Analysis: A. Formal Analysis; (3) Manuscript Preparation: A. Writing–Original Draft, B. Writing–Review and Editing; (4) A. Project Administration, B. Funding Acquisition, C. Resources, D. Supervision.

J.K.B.: 1A, 1B, 1C, 1D, 1E, 1F, 2A, 3A, 4A.

R.L.P.: 1B, 1C, 1E, 3B.

J.G.V.H.: 1B, 1E, 3B.

J.L.B.: 1B, 1D, 3B.

J.K.: 1B, 1D, 3B.

J.R.: 1B, 3B.

V.M.: 1D, 3B, 4A.

L.K.F.: 1D, 3B, 4A.

M.G.: 1B, 3B.

J.V.: 1D, 3B, 4A.

G.‐H.S.: 3B, 4C.

K.F.: 3B, 4C.

P.K.: 3B, 4C.

A.A.K.: 1A, 1B, 3A, 4B, 4C, 4D.

## Financial Disclosures

J.K.B. is a fellow of the BIH‐Charité Junior Clinician Scientist Programme funded by the Charité‐Universitaetsmedizin Berlin and Berlin Institute of Health (BIH) and is supported by the Deutsche Forschungsgemeinschaft (DFG, German Research Foundation) Project‐ID 424778381–TRR 295. R.L.P. None. J.G.V.H. is a fellow of the BIH‐Charité Junior Clinician Scientist Programme funded by the Charité‐Universitaetsmedizin Berlin and Berlin Institute of Health (BIH). J.L.B. is a fellow of the BIH‐Charité Junior Clinician Scientist Programme funded by the Charité‐Universitaetsmedizin Berlin and Berlin Institute of Health (BIH) and is supported by the Deutsche Forschungsgemeinschaft (DFG, German Research Foundation) Project‐ID 424778381–TRR 295. J.K. is supported by the Deutsche Forschungsgemeinschaft (DFG, German Research Foundation) Project‐ID 424778381–TRR 295. J.R. is a fellow of the BIH‐Charité Junior Clinician Scientist Programme funded by the Charité‐Universitaetsmedizin Berlin and Berlin Institute of Health (BIH) and has received speaker honoraria from Medtronic. V.M. is supported by the Lundbeck Foundation as part of the collaborative project grant “Adaptive and precise targeting of cortex‐basal ganglia circuits in Parkinson's Disease” (Grant No. R336‐2020‐1035). L.K.F. is a fellow of the BIH‐Charité Junior Clinician Scientist Programme funded by the Charité‐Universitaetsmedizin Berlin and Berlin Institute of Health (BIH) and has received speaker honoraria from Medtronic. M.G. None. J.V. is supported by a Doctoral Research Grant from the German Academic Exchange Service (Deutscher Akademischer Austauschdienst, DAAD). G.‐H.S. is supported by the Deutsche Forschungsgemeinschaft (DFG, German Research Foundation) Project‐ID 424778381–TRR 295 and has received honoraria from Medtronic and Boston Scientific. K.F. is supported by the German Federal Ministry of Research and Education (BMBF): FKZ 13GW0395 and Berlin Institute of Health (BIH): FKZ BIH_PRO_634. P.K. received speaker honoraria from Stadapharm and Medtronic and was in the Advisory Board of Medtronic, AbbVie and Gerresheimer. A.A.K is supported by the Deutsche Forschungsgemeinschaft (DFG, German Research Foundation) Project‐ID 424778381–TRR 295 and under Germany's Excellence Strategy–EXC‐2049–390,688,087 (Project Neurocure–BrainLab) and is supported by the Lundbeck Foundation as part of the collaborative project grant “Adaptive and precise targeting of cortex‐basal ganglia circuits in Parkinson's Disease” (Grant No. R336‐2020‐1035). She has served on advisory boards of Medtronic and has received honoraria and travel support from Medtronic, Boston Scientific, Ipsen Pharma, and Teva.

## Supporting information


**Data S1.** Supporting Information.

## Data Availability

The data that support the findings of this study are available from the corresponding author upon reasonable request.
