# Supplementary material for: Long‐Term Stability of Spatial Distribution and Peak Dynamics of Subthalamic Beta Power in Parkinson's Disease Patients
Source: Mov Disord. 2025 Mar 18;40(6):1070–84. doi: 10.1002/mds.30169 (PMC12160969; doi:10.1002/mds.30169)
Supplement: Supplementary file 1 — Data S1. Supporting Information. [file MDS-40-1070-s001.docx]

Long-term stability of spatial distribution and peak dynamics of subthalamic beta power in Parkinson’s disease patients

Supplementary material

**MATERIALS AND METHODS**

**ECG artifacts:**

**
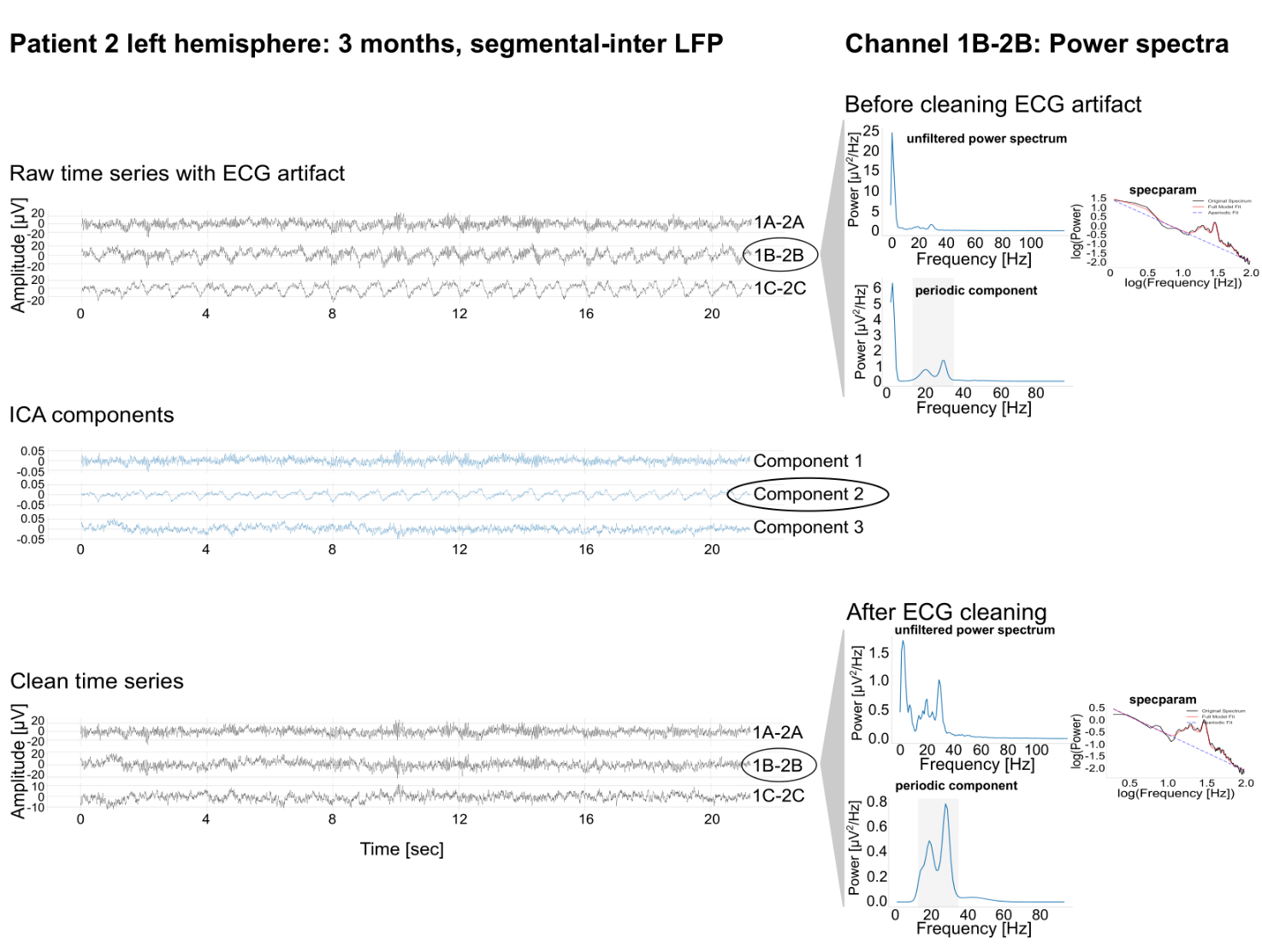
**

**Supplementary Figure S1. ECG artifact cleaning.** Example of a visually detected ECG artifact in the time series of patient 2, left hemisphere, segmental-inter channel group. The panels on the left side show the time series of three channels with an artifact in channels 1B-2B and 1C-2C at the top, three ICA components of the LFP signals in the middle, and the three channels after removing ICA component 2 from the time series at the bottom. The panels on the right side show the corresponding power spectra of channel 1B-2B before (top) and after (bottom) removing the ECG component. Power spectra are depicted as raw and unfiltered spectra at the top and as the periodic component after running the described *specparam* model at the bottom.

***specparam* parameters:**

For each computed power spectrum a *specparam* model [1] was fitted over a frequency range of 2-95 Hz using following parameters: peak_width_limits = [3, 20.0], max_n_peaks = 4, min_peak_height = 0.1, aperiodic_mode = ”fixed”. Power spectra were fitted without respecting a knee in the aperiodic component, since recent studies have shown the absence of a knee in the aperiodic component in the STN [2]. Identified peak parameters (CF, power) were extracted. From 66 DBS leads, the total number of fitted channels was 2376. The mean ± SEM of the *specparam* error was 0.076 ± 0.0003 and of the *specparam* r squared was 0.967 ± 0.0005. See Supplementary Figure S1 and S2 for example power spectra fitted with the described *specparam* model to extract only the periodic component of spectral power for further analysis.


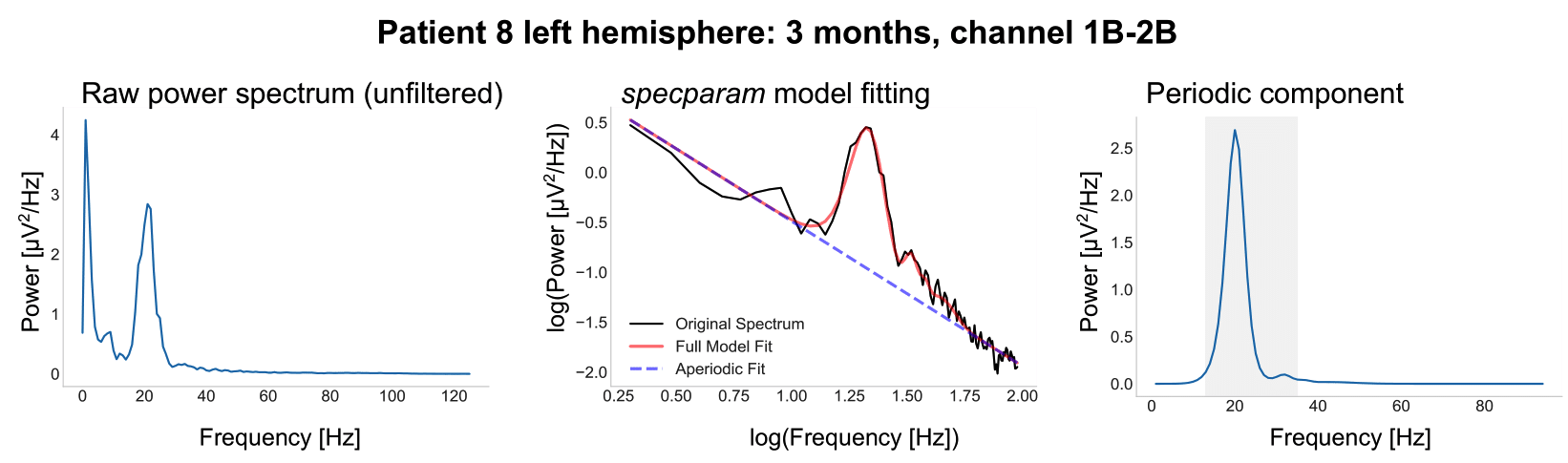


**Supplementary Figure S2. *specparam*** **LFP processing.** Example of the processing of a single power spectrum from channel 1B-2B of the left hemisphere of patient 8, three months post-surgery. The unfiltered power spectrum (left panel) was fitted with the described *specparam* model (mid panel) and only the periodic component of the fitted power spectrum was used for the analysis (right panel).

**Estimation of pseudo-monopolar beta power**

We developed a method that weights spectral power based on the relationship between the amplitude of a signal scaling with the inverse of the squared distance between the contact of interest and the central position of each recording [3]. The sum of all weighted LFPs resulted in a pseudo-monopolar beta power estimate corresponding to a segmented contact. When only analyzing power corresponding to segmented contacts, we included all available bipolar LFPs from the segmented contacts (in total *n=9* LFPs), hence all channels from the segmental-1L and segmental-2L groups. For analyses also including power corresponding to ring contacts, we included all bipolar LFPs (including ring LFP, in total *n=12* LFPs). The Euclidean coordinates of each contact are based on a 2 mm distance between the center of each contact level and a radius of 0.65 mm (Suppl. Table S1).

First, for each bipolar channel the mean Euclidean coordinates of their two involved recording partners were calculated. This provided a pseudo-bipolar recording site in the center of each bipolar channel, from which the distance *(d)* to the screened contact was calculated. To generate a pseudo-monopolar estimate corresponding for instance to the segmented contact 1B, the distance of each bipolar channel to contact 1B was calculated. The distance calculation followed the formula below, derived from the Pythagorean theorem, e.g., for the contact 1B, where *i* is a bipolar channel (Suppl. Table S2):

$$d_{i}= \sqrt{({\left( x_{1B}-x_{i} \right)^{2}+\left( y_{1B}-y_{i} \right)}^{2}+{(z_{1B}- z_{i})}^{2})}$$

Second, the spectral power from each pseudo-bipolar recording site was weighted depending on that distance to the screened contact (e.g., 1B). To be precise, each power spectrum was multiplied by its inverse squared distance between its pseudo-bipolar recording site and the screened contact (e.g., 1B). Finally, the sum of all weighted power spectra from all included bipolar channels (*n*) yielded a single power spectrum estimating the pseudo-monopolar power of an individual segmented contact (e.g., 1B), as summarized by the following equation:

$${PSD}_{1B}= \sum_{i=1}^{n} {PSD}_{i}* \frac{1}{{d_{i}}^{2}}$$

In this formula, *PSD_i_* represents the spectral power of a bipolar channel *i* from all included channels (*n*). *d_i_* is the distance between the Euclidean coordinates of the pseudo-bipolar recording site of channel *i* and the contact of interest. Beta power was calculated by averaging the power in the beta range (13-35 Hz).

| **Contact** | **X** | **Y** | **Z** |
| --- | --- | --- | --- |
| Segmented Contacts |  |  |  |
| 1A | 0.650 | 0.000 | 2.0 |
| 1B | -0.325 | 0.563 | 2.0 |
| 1C | -0.325 | -0.563 | 2.0 |
| 2A | 0.650 | 0.000 | 4.0 |
| 2B | -0.325 | 0.563 | 4.0 |
| 2C | -0.325 | -0.563 | 4.0 |
| Ring Contacts |  |  |  |
| 0 | 0.000 | 0.000 | 0.0 |
| 1 | 0.000 | 0.000 | 2.0 |
| 2 | 0.000 | 0.000 | 4.0 |
| 3 | 0.000 | 0.000 | 6.0 |

**Supplementary Table S1.** Euclidean x-, y-, z-coordinates of the center of each contact (segmented or ring). All values are reported in millimeters.

| **Contact** | **Bipolar Channel** | **X** | **Y** | **Z** | **Distance** |
| --- | --- | --- | --- | --- | --- |
| 1B | 1A2A | 0.650 | 0.000 | 3.0 | 1.51 |
| 1B | 1B2B | -0.325 | 0.563 | 3.0 | 1.00 |
| 1B | 1C2C | -0.325 | -0.563 | 3.0 | 1.51 |
| 1B | 1A1B | 0.162 | 0.281 | 2.0 | 0.56 |
| 1B | 1B1C | -0.325 | 0.000 | 2.0 | 0.56 |
| 1B | 1A1C | 0.162 | -0.281 | 2.0 | 0.97 |
| 1B | 2A2B | 0.162 | 0.281 | 4.0 | 2.08 |
| 1B | 2B2C | -0.325 | 0.000 | 4.0 | 2.08 |
| 1B | 2A2C | 0.162 | -0.281 | 4.0 | 2.22 |

**Supplementary Table S2.** For an exemplary segmented contact 1B the Euclidean distances to bipolar segmental channels, along with their central x-, y-, z-coordinates are illustrated. All values are reported in millimeters.


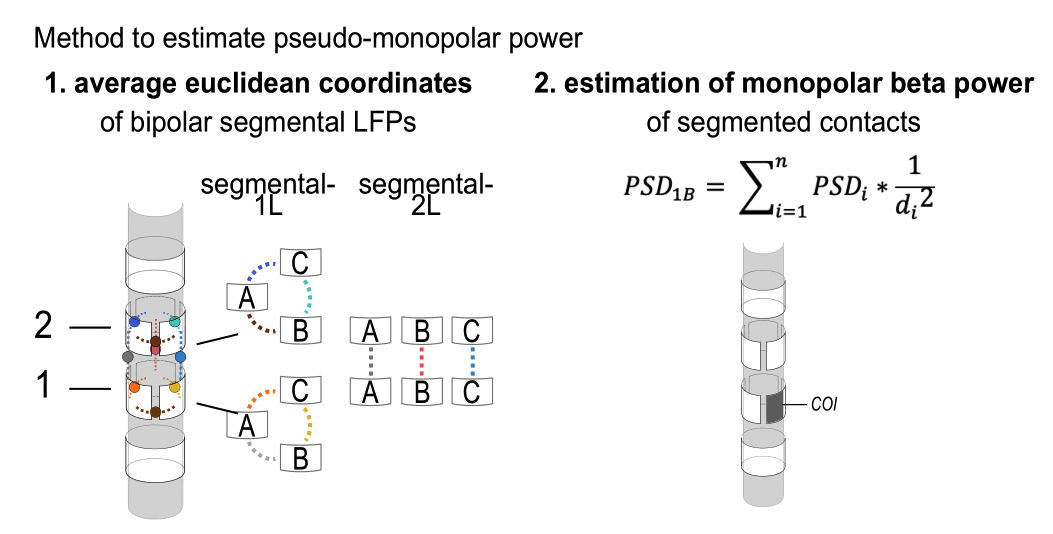


**Supplementary Figure S3.** **Pseudo-monopolar power estimation aligned to individual contacts.** For instance, for calculating pseudo-monopolar power at contact 1B, we first determined the average Euclidean coordinates of each pair of LFP recording contacts – defining a pseudo-bipolar recording site (colored dots) - and then calculated the distance to the contact of interest (COI). Second, we calculated the spectral power as a sum over weighted (inverse squared distance) power of each bipolar recording (Pb), yielding a single estimated pseudo-monopolar power spectrum (Pm) for the example contact 1B.

**Statistics**

***Longitudinal analysis of absolute peak frequency shifts (Suppl. Fig. S5)***

Mann-Whitney-U-Tests assessed absolute changes in peak frequencies between sessions in all selected ring channels of hemispheres with consistent pairs of recording sessions.

***Inclusion criteria for each analysis***

*Peak parameter analysis:*

- **Large cohort analysis of peak CF and power (Fig. 2A):**

Included hemispheres with two consistent recording sessions (0-mo and 3- or 12-mo FU) and identified peaks in both sessions to ensure consistency. Channel selection and fixed frequency ranges were based on either session 3- or 12-mo FU. In all other peak parameter analyses, channel selection and fixed frequency ranges were based only on session 3-mo FU.

- **Directional longitudinal analysis of peak power (Fig. 2C) and peak CF (Suppl. Fig. S6 B):** Included hemispheres with three consistent recording sessions and identified peaks in all three sessions to ensure consistency.
- **Longitudinal analysis of substantial peak frequency shifts >5.0 Hz (Fig. 2D) or >2.5 Hz (Suppl. Fig. S6 A):** Included hemispheres with three consistent sessions and at least one identified peak in two consecutive sessions. Peak frequency shifts (>2.5 or >5 Hz) or missing peaks were classified as substantial shifts due to their potential impact on aDBS performance.
- **Longitudinal analysis of absolute peak frequency shifts (Suppl. Fig. S5):** Included hemispheres with consistent pairs of recording sessions with an available 3-mo FU session and identified peaks in both sessions.

*Spatial distribution analysis:*

- **Correlation of bipolar beta power across 12 channels (Fig. 3A):**

Included all hemispheres with three consistent recording sessions.

- **Longitudinal analysis of vertical and horizontal shifts of maximal beta power (Fig. 3B-C):** Included all hemispheres with consistent pairs of recording sessions. Each period (e.g., 0-3-mo FU) was tested independently.
- **Beta power distribution across active versus inactive contacts (Fig. 3D):** Included all hemispheres after the first optimization of DBS settings at 3-mo FU, regardless of session consistency. Excluded hemispheres with bipolar (*n=2* STN) or interleaving (*n=3* STN) stimulation programs. Each FU session was tested independently.

**Software**

LFP data was converted using MATLAB R2023a v9.14.0 and the open source “Perceive” toolbox (https://github.com/neuromodulation/perceive). Recordings were stored in a standardized data structure to ensure file loading using the open-source repository “PyPerceive” in Python programming language (https://github.com/jgvhabets/PyPerceive). For all analyses custom-written Python scripts and following software packages were used: Python v3.11.3 [4], numpy v1.25.0 [5], pandas v2.0.3 [6], matplotlib v3.7.1 [7], scipy v1.10.1 [8], seaborn v0.11.1 [9], mne v1.4.2 [10] and fooof v1.0.0 [1]. For statistical purposes following packages were used: pingouin v0.5.3 [11], seaborn, sklearn [12] and statsmodel v0.14.0.

**
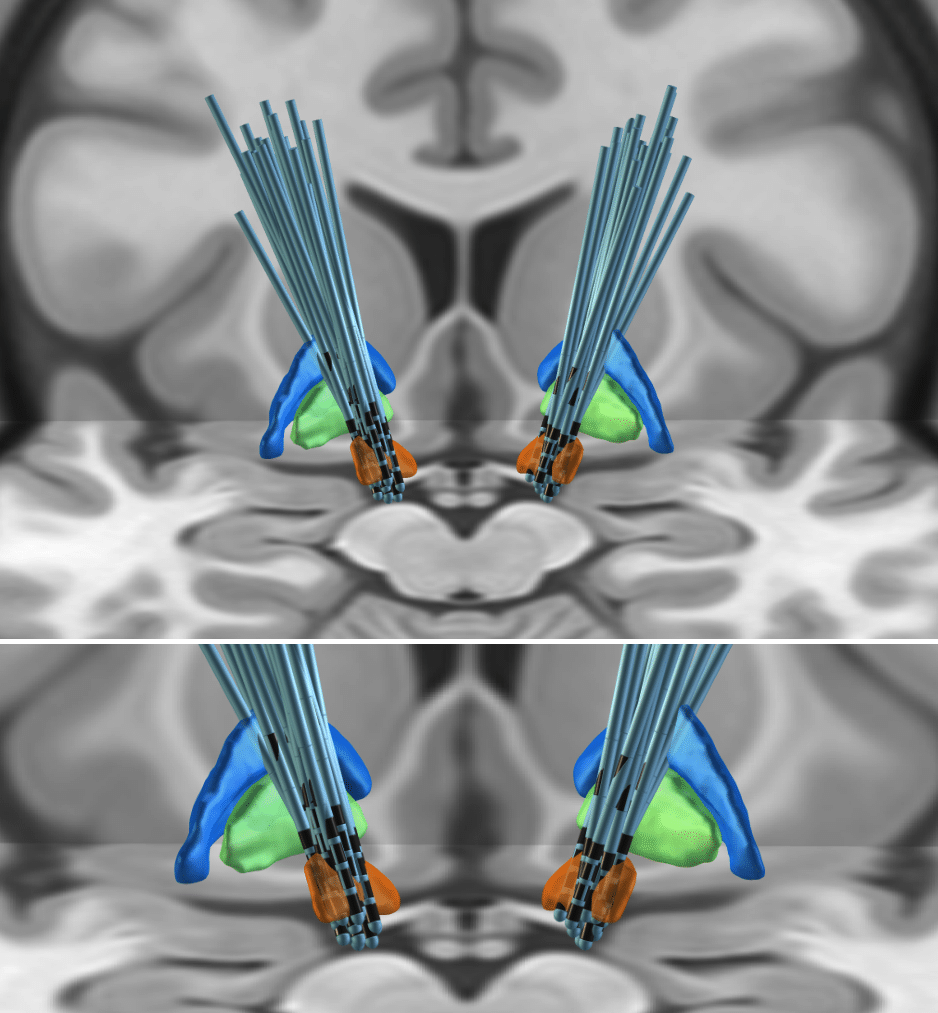
**

**Supplementary Figure S4. Lead Localizations.** DBS electrodes (*n=66*) were localized and normalized into the Montreal Neurological Institute (MNI) space (2009b, nonlinear, assymmetric) using registration approaches as implemented in Lead-DBS [13]. View from posterior. The STN is illustrated in orange, the internal pallidum in green and the external pallidum in blue.

| **Count of subjects (N)** | **“Large cohort” (sessions 0+3/12)** | **“Early” (sessions 0+3+12)** | **“Late” (sessions 3+12+>18)** | **“Long-term cohort” (sessions 0+3+12+>18)** |
| --- | --- | --- | --- | --- |
| 1 | 4 | 4 | 1 | 4 |
| 2 | 5 | 5 | 2 | 6 |
| 3 | 6 | 6 | 3 | 8 |
| 4 | 7 | 8 | 4 | 9 |
| 5 | 8 | 9 | 6 | 22 |
| 6 | 9 | 22 | 8 |  |
| 7 | 10 | 24 | 9 |  |
| 8 | 11 | 25 | 12 |  |
| 9 | 13 | 26 | 14 |  |
| 10 | 16 | 28 | 15 |  |
| 11 | 17 | 29 | 19 |  |
| 12 | 18 | 30 | 22 |  |
| 13 | 20 | 31 |  |  |
| 14 | 21 | 32 |  |  |
| 15 | 22 | 33 |  |  |
| 16 | 23 |  |  |  |
| 17 | 24 |  |  |  |
| 18 | 25 |  |  |  |
| 19 | 26 |  |  |  |
| 20 | 27 |  |  |  |
| 21 | 28 |  |  |  |
| 22 | 29 |  |  |  |
| 23 | 30 |  |  |  |
| 24 | 31 |  |  |  |
| 25 | 32 |  |  |  |
| 26 | 33 |  |  |  |

**Supplementary Table S3. Participants’ sub-ID included in four subgroups.** Large cohort with recordings at session 0-mo and at either 3- or 12-mo FU (*n=26* patients), subgroup with consistent “early” sessions 0-, 3- and 12-mo FU (*n=15* patients), subgroup with consistent “late” sessions 3-, 12- and >18-mo FU (*n=12* patients) and subgroup with all four sessions (*n=5* patients).

**RESULTS**

***Absolute CF shifts from hemispheres with consistent pairs of sessions***

The CF of the highest peak within the beta band changed notably during the first three months (0-3) and stabilized in most hemispheres during the last two periods (3-12 and 12-18/24) (Suppl Fig. S5, upper panel). Absolute peak CF shifts were greater between 0- and 3-mo FU (mean ± SD, respectively: 5.89 Hz ± 4.68, *n=37*) than between 3- and 12-mo FU (3.67 Hz ± 4.67, *n=42*, *p=0.008* MWU*;* Suppl. Fig. S5, Suppl. Table S3). In hemispheres with double beta peaks (Fig. 2B), large peak frequency shifts occurred when the highest beta peak switched between low- and high-beta peaks over time.

The peaks in either the low- (13-20 Hz) or high-beta band (21-35 Hz) separately revealed milder CF shifts, averaging less than 2 Hz in the low beta and 2-3 Hz for high beta peaks with no significant differences across time (p≥0.05) (Suppl. Fig. S5, Suppl. Table S3).

| **Ring LFP group** | **0-3** | **3-12** | **12-18/24** |
| --- | --- | --- | --- |
| Full beta range | 5.89 ± 4.68, *n=37* | 3.67 ± 4.67, *n=42* | 4.17 ± 4.99, *n=23* |
| Low-beta range | 1.61 ± 1.43, *n=22* | 1.56 ± 1.23, *n=28* | 1.63 ± 1.43, *n=9* |
| High-beta range | 2.79 ± 0.99, *n=31* | 2.89 ± 1.74, *n=39* | 2.28 ± 2.24, *n=23* |

**Supplementary Table S4. Peak frequency shifts in full beta, low-beta and high-beta ranges.** Absolute peak frequency shifts between sessions (mean [Hz] ± SD). N refers to the number of hemispheres with detected peaks in both sessions of each session comparison (0-3, 3-12 and 12-18/24).


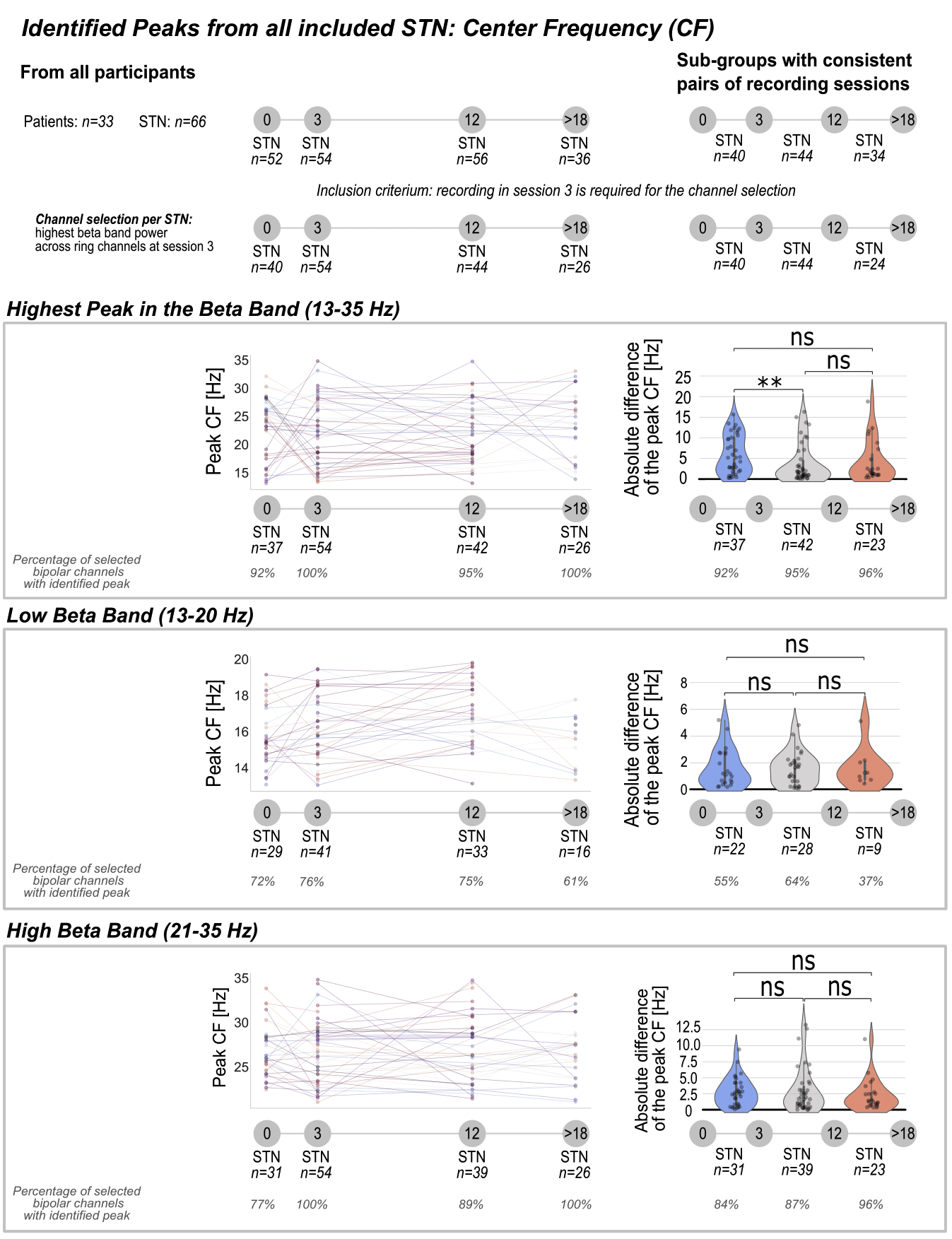


**Supplementary Figure S5. Center frequency (CF) of all identified peaks using *specparam*.** From a total of *n=66* STN, only hemispheres with an available 3-mo FU session were included to enable ring channel selection based on maximal beta power. Left: CFs of all identified peaks are shown across the beta, low-beta and high-beta ranges. Right: For hemispheres with consistent pairs of recording sessions, the absolute differ-ences in peak CFs between sessions (0-3, 3-12 and 12- >18-mo FU) were compared using Mann-Whitney-U-Tests. Sample sizes and percentages of channels with identified peaks are given below.

***Peak shifts above threshold or peak disappearance***

Peak CF shifts exceeding thresholds of 2.5 (Suppl. Fig. S6 A) or 5.0 Hz (Fig. 2D), were quantified for all beta ranges, regardless of direction, including hemispheres with missing peaks in single sessions (classified as CF shift above the threshold). At least one peak in two compared sessions were identified in 100% in the full beta and high-beta ranges for both “early” (*n=*30 STN, sessions 0, 3, 12) and “late” subgroups (*n=*24 STN sessions 3, 12, >18). In the low-beta band, at least one peak was identified in 87 % of hemispheres in the “early” subgroup (*n=26* STN) and 79 % in the “late” subgroup (*n=19* STN) (Suppl. Fig. S6). We performed a Wilcoxon signed-rank test to compare binomial classifications of shifts (below or above the threshold) across two periods, e.g., sessions 0-3 vs. sessions 3-12.

Shifts >2.5 Hz were more common but followed similar trends as described for shifts >5 Hz (Fig. 2D). Significant shifts of the largest beta peak >2.5 Hz were more frequent during the earliest period (0-3-mo FU; *73 %*) compared to the subsequent period (3-12-mo FU; *43 %, p=0.029*). In contrast, no significant differences in CF shifts were found in the “late” subgroup between period 3-12-mo FU (*37 %*) and period 12- >18-mo FU (*37 %, p=1.0*, Suppl. Fig. S6 A).

The number of substantial shifts of low-beta and high-beta peaks >2.5 Hz did not differ significantly across time in neither the “early” nor “late” subgroups. Shifts >2.5 or peak disappearance occurred between sessions 0- and 3-mo FU in 54 % of low-beta peaks and from 63 % of high-beta peaks, while between 3- and 12-mo FU in 38 % and 50 %, respectively. In the “late” subgroup between 3- and 12-mo FU substantial frequency shifts occurred in 47 % of low-beta peaks and 46 % of high-beta peaks, while between 12- >18, in 58 % of low-beta peaks and 33 % of high-beta peaks.


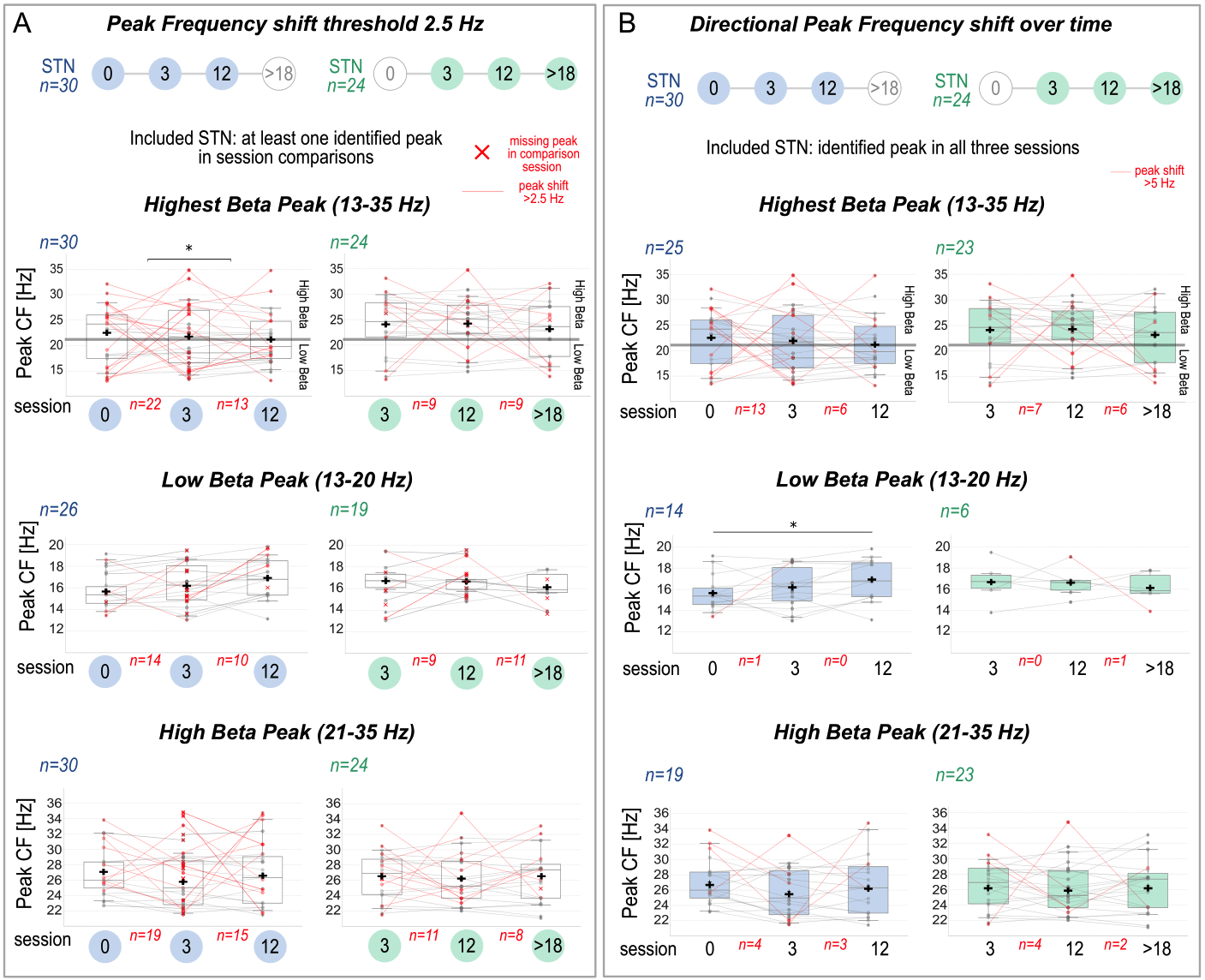


**Supplementary Figure S6. Center frequency (CF) shifts.** (A, B) Subgroup analyses of one selected channel per STN with consistent recording sessions: blue (0-3-12), green (3-12- >18). Inclusion of channels with consistently identified peaks in (A) at least one identified peak per session comparison or (B) in all sessions. Peak frequencies are illustrated for each session in the full beta (top), low-beta (middle) and high-beta band (bottom). Lines connect peaks from the same hemisphere. Red lines highlight CF shifts >2.5 (A) or >5 Hz (B). Only in A, missing peaks are marked with a red cross at the compared peak CF from the other session. The number of channels with significant CF shifts (>2.5 Hz) or peak disappearance is shown below in red (e.g., of 30 hemispheres, the largest beta peak shifted >2.5 Hz between sessions 0- and 3-mo FU in *n=22* hemispheres). The grey line at 21 Hz in the top panel marks the border between the low- and high-beta boundary. Black crosses in boxplots indicate the mean.


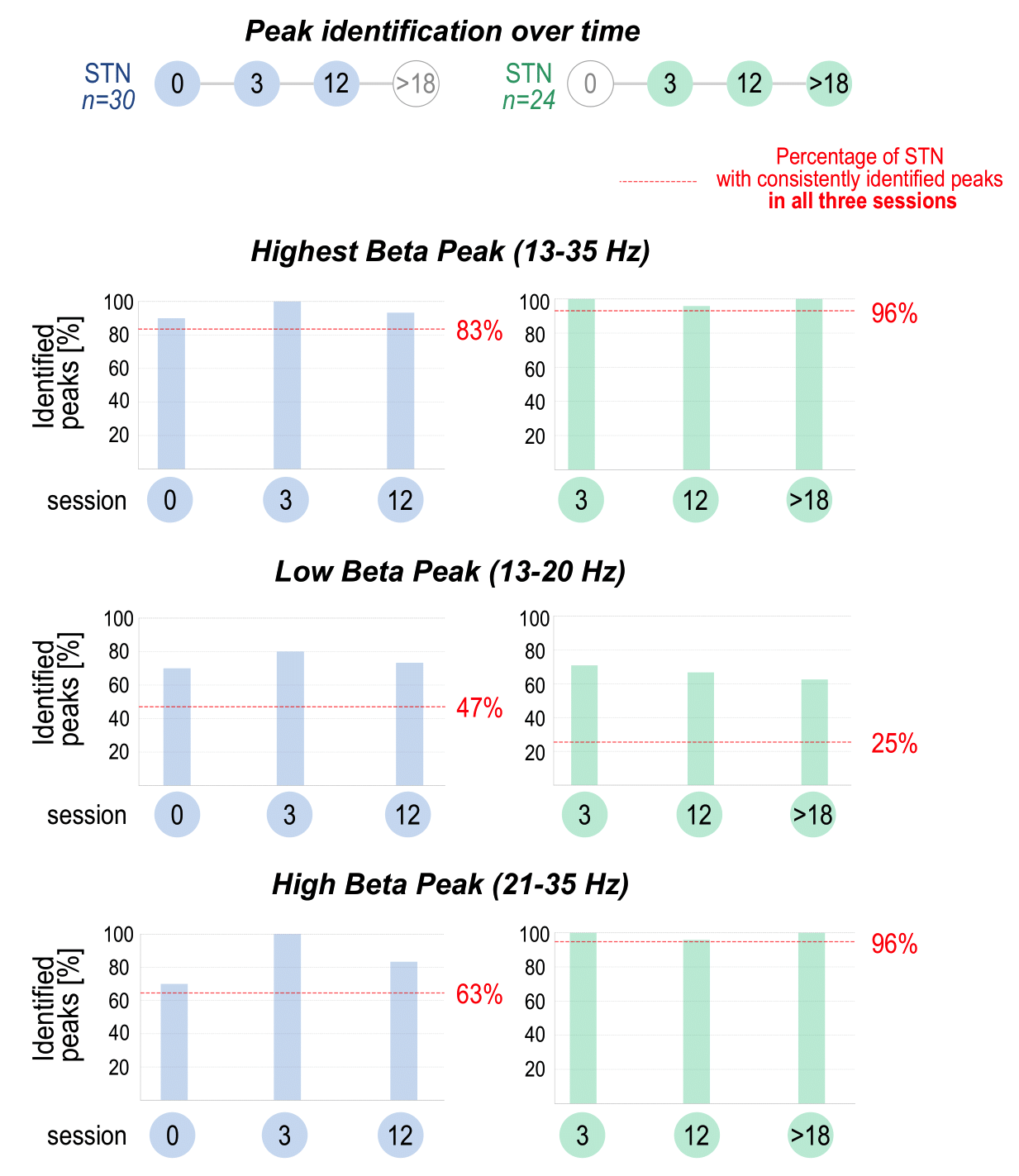


**Supplementary Figure S7. Longitudinal consistency of peak identification.** In both subgroups with three consistent recording sessions 0-3-12-mo FU (blue, *n=30)* and 3-12- >18-mo FU (green, *n=24*) the highest beta and high-beta peaks showed greater consistency in detection (using specified *specparam* parameters) compared to low-beta peaks over time. The percentage of STN with identified peaks is presented for each session, alongside the percentage of STN with consistently identified peaks in all three sessions within each subgroup (red dashed lines), across the different beta frequency ranges.

**REFERENCES**

1. Donoghue, T., et al., *Parameterizing neural power spectra into periodic and aperiodic components.* Nat Neurosci, 2020. **23**(12): p. 1655-1665.

2. Bush, A., et al., *Broadband aperiodic components of local field potentials reflect inherent differences between cortical and subcortical activity.* bioRxiv, 2023.

3. Buzsaki, G., C.A. Anastassiou, and C. Koch, *The origin of extracellular fields and currents--EEG, ECoG, LFP and spikes.* Nat Rev Neurosci, 2012. **13**(6): p. 407-20.

4. Perez, F. and B.E. Granger, *IPython: A system for interactive scientific computing.* Computing in Science & Engineering, 2007. **9**(3): p. 21-29.

5. van der Walt, S., S.C. Colbert, and G. Varoquaux, *The NumPy Array: A Structure for Efficient Numerical Computation.* Computing in Science & Engineering, 2011. **13**(2): p. 22-30.

6. McKinney, W.a.o., *Data structures for statistical computing in python*, in *Proceedings of the 9th Python in Science Conference*. 2010. p. 51-56.

7. Hunter, J.D., *Matplotlib: A 2D graphics environment.* Computing in Science & Engineering, 2007. **9**(3): p. 90-95.

8. Virtanen, P., et al., *SciPy 1.0: fundamental algorithms for scientific computing in Python.* Nat Methods, 2020. **17**(3): p. 261-272.

9. Waskom, M.L., *seaborn: statistical data visualization.* Journal of Open Source Software, 2021. **6**: p. 3021.

10. Gramfort, A., et al., *MEG and EEG data analysis with MNE-Python.* Front Neurosci, 2013. **7**: p. 267.

11. Vallat, R., *Pingouin: statistics in Python.* The Journal of Open Source Software, 2018. **3**: p. 1026.

12. Pedregosa, F., et al., *Scikit-learn: Machine Learning in Python.* Journal of Machine Learning Research, 2011. **12**: p. 2825-2830.

13. Horn, A., et al., *Lead-DBS v2: Towards a comprehensive pipeline for deep brain stimulation imaging.* Neuroimage, 2019. **184**: p. 293-316.
